# Recombination in sarbecovirus lineage and mutations/insertions in spike protein are linked to the emergence and adaptation of SARS-CoV-2

**DOI:** 10.6026/97320630018951

**Published:** 2022-10-31

**Authors:** Anup Som, Amresh Kumar Sharma, Priyanka Kumari

**Affiliations:** 1 Centre of Bioinformatics, Institute of Interdisciplinary Studies, University of Allahabad, Prayagraj - 211002, India

**Keywords:** Coronavirus, SARS-CoV-2, molecular phylogeny, recombination, codon adaptation index, spike protein, structural modeling

## Abstract

The outbreak of severe acute respiratory syndrome coronavirus 2 (SARS-CoV-2) in Wuhan city, China in December 2019 and thereafter its spillover across the world has created a global pandemic and public health crisis. Right after, there has been intense interest in understanding how the SARS-CoV-2 originated and evolved. This paper also aims to shed light on the origin and evolution of SARS-CoV- 2. A consensus result based on whole genome phylogeny, gene tree analysis, and genetic similarity study revealed that SARS-CoV-2 evolved from Bat-CoV-RaTG13. Furthermore, recombination analysis indicated that probable origin of SARS-CoV-2 is the results of ancestral intra-species recombination events between bat coronaviruses belonging to Sarbecovirus sub-genus. Multiple sequence alignment (MSA) revealed the insertion of four amino acid residues "PRRA" (Proline-Arginine-Arginine-Alanine) to the S1/S2 site in the spike protein of SARS-CoV-2, and structural modeling of spike protein of bat-CoV-RaTG13 also shows a high number of mutations at one of the receptor binding domains (RBD). Acquisition of the furin cleavage sites ("PRRA") along with high number of mutations at one of its RBD is probably responsible for the adaptation of SARS-CoV-2 into human systems. Furthermore, the codon adaptation index (CAI) was used to quantify the magnitude of adaptive efficacy of SARS-CoV-2 in human host in comparison with SARS-CoV. The CAI result showed a relatively less adaptive efficacy of the newly emerged SARS-CoV-2 to the human systems, which might be an indication of its mild clinical severity and progression compared to SARS-CoVs.

## Background:

Coronaviruses are single-stranded RNA viruses of 26 to 32 kilo bases (Kb) nucleotide chain and consist of both structural and non-structural proteins. They have been known to cause lower and upper respiratory diseases, central nervous system infection and gastroenteritis in a number of avian and mammalian hosts including humans [1-2]. The recent outbreak of novel coronavirus (SARS-CoV-2) associated with acute respiratory disease called coronavirus disease 19 (commonly known as COVID-19) has caused a global pandemic. As of 30th June 2022, more than 551 million laboratories confirmed COVID-19 cases and approximately 6.34 million people have died and further COVID-19 appears as a global threat to public health as well as to the human civilization as economic and social disruption caused by the pandemic is devastating (WHO, COVID-19 situation reports). Coronaviruses are placed within the family Coronaviridae, which has two subfamilies namely Ortho coronavirinae and Torovirinae. Ortho coronavirinae has four genera: Alpha coronavirus (average genome size 28kb), Betac oronavirus (average genome size 30kb), Gamma coronavirus (average genome size 28kb), and Delta coronavirus (average genome size 26kb) [3]. Coronaviruses are typically harbored in mammals and birds. Particularly Alpha coronavirus and Beta coronavirus infect mammals, and Gamma coronavirus and Delta coronavirus infect avian species [4-6]. SARS-CoV-2 is a member of the genus Beta coronavirus and subgenus Sarbecovirus. Figure 1(see PDF) depicts the taxonomical classification of SARS-CoV-2. The previous important outbreaks of coronaviruses are severe acute respiratory syndrome coronavirus (SARS-CoV or SARS-CoV-1) outbreak in China in 2002/03, Middle East respiratory syndrome coronavirus (MERS-CoV) outbreak in 2012 that resulted severe epidemics in the respective geographical regions [7-9]. The present outbreak of SARS-CoV-2 is the third documented spillover of an animal coronavirus to humans in only two decades that has resulted in a major pandemic [10-12].

Since COVID-19 started, there has been intense research on the origin and evolution of the SARS-CoV-2, which resulted a very large number of publications on the origin and evolution of SARS-CoV-2. The key reported findings, out of the large number of research outcomes, are: bat and/or pangolin are the natural reservoir of SARS-CoV-2, Bat-CoV-RaTG13 is the closest relative of SARS-CoV-2, transmission of SARS-CoV-2 to human population took place via intermediate hosts, and mutations in the furin cleavage site in spike protein probably linked with the adaptation to the human systems etc. However significant progress has been made towards understanding the origin, transmission and adaptation mechanism of SARS-CoV-2 but the exact origin, cause of emergence and infection mechanism of SARS-CoV-2 are yet to be fully known. Therefore, as more-and-more datasets are generating, there are needs for further in-depth studies on the emergence of SARS-CoV-2.

## Materials and Methods:

### Data selection:

162 Orthocoronavirinae genomes were retrieved from NCBI (https://www.ncbi.nlm.nih.gov/), Virus Pathogen Database and Analysis Resource (https://www.viprbrc.org/). We only considered complete genome sequences having no unidentified nucleotide characters. Our datasets included 23 Alpha coronavirus, 92 Beta coronavirus, 32 Delta coronavirus and 15 Gamma coronavirus genomes belonging to different subgenus, diverse host species and from wide geographical location. Further for rooting the tree, we used two genome sequences from Torovirus and two from Bafinivirus belonging to domestic cow and fish respectively. The genera Torovirus and Bafinivirus belong to the sub-familyTorovirinave of the family Coronaviridae. Overall, the phylogenetic analysis consists of 166 complete viral genomes (162 Ortho coronavirinae and four Torovirinave genomes).

### Phylogenetic reconstruction:

The genome sequences were aligned using the MAFFT alignment tool [13]. Genome tree of the Ortho coronavirinae and Beta coronaviruses were reconstructed using maximum likelihood (ML) method and GTR+G+I model of nucleotide substitution as revealed by the model test with1000 boots trap support. The model test was performed for accurate phylogenetic estimation by using Model Finder, which is implemented in IQ-TREE version 1.5.4 [14]. Phylogenetic trees were reconstructed using IQ-TREE software [15]. The trees were visualized with iTOL tool [16]. Five gene trees of the Beta coronaviruses were reconstructed using Orf1ab, Spike (S), Envelope (E) Membrane (M), and Nucleocapsid (N) amino acid sequences. The ML method of tree reconstruction and protein-specific amino acids substitution model as revealed by Model Finder was used for gene tree reconstruction. Bootstrap test with 1000 bootstrap replicates was carried out to check the reliability of the gene trees.

### Genome and gene recombination analysis:

Potential recombination events in the history of the Beta coronaviruses were assessed using the RDP5 package [17]. The RDP5 analysis was conducted based on the complete genome sequence using RDP, GENECONV, BootScan, MaxChi, Chimera, SiScan, and 3Scan methods. Putative recombination events were identified with a Bonferroni corrected P-value cut-off of 0.05 supported by more than four methods.

### Sequence and structural analysis:

The homology and genetic variations analysis of sequences in different genomic regions of SARS-CoV-2 strain Wuhan Hu-01 (MN908947) is compared to bat-CoV-RaTG13 (MN996532) and pangolin-CoV-GX-P5E (MT040336) using CLUSTAL W (https://www.genome.jp/tools-bin/clustalw) and multiple sequence alignment (MSA) analysis of spike proteins were performed using CLUSTAL OMEGA (https://www.ebi.ac.uk/Tools/msa/clustalo/). The structures of the spike protein of SARS-CoV-2 Wuhan Hu-1 (PDB: 6XLU), bat-CoV-RaTG13 (PDB: 6ZGF) were retrieved from PDB database [18]. The spike protein for pangolin coronavirus was not available so it was modeled using SWISS-MODEL SERVER (https://swissmodel.expasy.org) with 6XR8 as template. These structures were compared using the structure superimposition/structure alignment tool of Chimera software [19].

## Results and Discussion:

This study aims to understand the origin and evolutionary trajectory of SARS-CoV-2 using molecular phylogenetic, genomic diversity, recombination, and structural analyses. Particularly, we carried out the phylogenetic study of human SARS-CoV-2 from their deep ancestral roots (i.e., from the sub-family (Ortho coronavirinae) to the lineage (SARS-CoV-2); Figure 1 - see PDF). Accordingly, the molecular phylogenetic analysis was based on two-stage; whole genome phylogeny followed by gene trees analyses. Firstly, reconstruction of genome phylogeny of the Ortho coronavirinae genomes and study the cladistic/evolutionary relationships of its four genera. Secondly, reconstruction of Beta coronavirus genome and gene phylogeny that included its five sub-genera namely Embecovirus, Hibecovirus, Merbecovirus, Nobecovirus and Sarbecovirus, and study the evolutionary relations of these five subgenera and find linage containing SARS-CoV-2.

## Ortho coronavirinae phylogeny:

The genome phylogeny of Ortho coronavirinae depicts that Alpha, Beta, Delta and Gamma coronaviruses clustered according to their cladistic relationships where Alpha coronavirus clade appeared as a basal radiation of the Ortho coronavirinae phylogeny (Figure 2 - see PDF). This result is consistent with the other studies [20, 21]. Furthermore, analysis of the clades found that Gamma coronavirus and Delta coronavirus clades are monophyletic (originated from a single common ancestor). This result is supported by their hosts' nature; as both types mostly infect avian species [22]. Further, a deeper analysis of the Ortho coronavirinae genome tree revealed that irrespective of their geographical locations, the host-specific strains are clustered together. This is probably due to the host-specific mutations, which is an important characteristic of viral genomes for their survival and replication [23-25]. For example, Alphacoronavirus strains from ferret_Japan and ferret_Netherland are monophyletic. Similarly cat_UK is monophyletic with cat_Netherland, and human_China is monophyletic with human_Netherland. Further analysis revealed all Alpha coronavirus camel strains of Saudi Arabia appeared in a distinct sub-clade where bat_Ghana strain appeared as outgroup which indicates interspecies transmission took place from bat_Ghana to camel. A number of scientific evidences, based on the independent datasets, also reported that coronavirus transmission took place to humans through intermediate hosts [26-29].

Delta coronavirus and Gamma coronavirus clades exhibit a similar evolutionary pattern. In case of Delta coronaviruses, swine_Vietnam and swine_Hong Kong shared a single common ancestor. Similarly, swine_China and swine_South Korea are monophyletic clade and swine_Japan is monophyletic with swine_South Korea. In case of Gamma coronaviruses (whose natural hosts are avian species), chicken_Peru and chicken_Uruguay are monophyletic. Similarly, chicken_Iraq is monophyletic with chicken_Egypt strain. These observations reconfirm that coronaviruses are present in a large number of hosts those are widespread in different geographical location and coronaviruses undergo host-specific mutation/adaptation. This is not surprising as sequences of corona viruses isolated from different geographical locations and found that genetic changes through recurrent mutations of the virus are continuously arising, which ultimately promote host adaptation [30,31].

## Beta coronavirus phylogeny:

Phylogenetic analysis of Beta coronavirus genomes revealed that the five sub genera clustered separately (Figure 3-see PDF). Furthermore, like other three genera, the Beta coronavirus genome tree depicts that the host-specific strains from distance geographical locations formed monophyletic clades. For example, in Embecovirus clade, strain BJ01_P9_human_China is monophyletic with Caen1_human_France strain. Similarly, Embecovirus B1_24F_buffalo_Bangladesh is monophyletic with BCV_AKS_01_cattle_China.

SARS-CoV-2 (isolated from human) belongs to Sarbecovirus sub-genus. Sarbecoviruses formed three distinct clades (Figure 3-see PDF), where Clade 1 consists of only bat as host species. InClade2, host species are bat, civet and human. Similarly, in Clade3 the host species are bat, pangolin and human and it depicts bat-CoV-RaTG13 (marked as MRCA in Figure 3-see PDF) is closest to the human SARS-CoV-2 as all human SARS-CoV-2s clustered in a clade, and formed a monophyletic clade with bat-CoV-RaTG13 strain. Clade 3 also shown that pangolin (PCoV-GX-P5E) is the second closest relative of human SARS-CoV-2. Further, deep node analysis of Clade 3, shows that human SARS-CoV-2s, pangolin CoVs (strains PCoV-GX-P4L/P3B/P1E/P5E/P2V) and bat-CoVs (strains bat-SL-CoVZXC21 and bat-SL-CoVZC45) shared a single common ancestor (Figure 3-see PDF). This observation suggests bat and pangolin is the natural host of SARS-CoV. The same inference had also been reported by a number of studies [24,25, 26,27,28, 29, 30,31, 32].Furthermore, phylogenetic analysis reveals that the MERS-CoVs, SARS-CoVs, SARS-CoV-2s are conserved in their respective hosts (e.g. all bat hosts clustered in Clade 2 and human hosts are in Clade 3). This observation led to the conclusion that host-specific mutations of MERS-CoVs, SARS-CoVs and SARS-CoV-2s occurred, which is probably to facilitate colonization and invade to the host immune system [26, 33, 34].

## Codon adaptation index analysis:

The codon adaptation index (CAI) was used to quantify the magnitude of adaptive efficacy of SARS-CoV-2 in human host in comparison with SARS-CoV. The CAI analysis was performed using the CAIcal server [35]. It was found that the average CAI value for SARS-CoV-2 with respect to human host is 0.692, which was considerably lower than for SARS-CoV (0.721). This result indicates a relatively less adaptive efficacy of the newly emerged SARS-CoV-2 to the human systems, which might be an indicative of its mild clinical severity and progression compared to SARS-CoVs. Further, in supports of this observation, we thoroughly review the existing scientific evidences on the host adaptation of SARS-Cov-2. A number of studies based on the CAI and relative synonymous codon usage (RSCU) reported that the host adaptation of SARS-CoV-2 occurred and probably this adaptation took place after SARS-CoV-2 diverge from RaTG13 because RaTG13 is less perfectly correlated with human cellular systems [27, 31,36].

## Average nucleotide identity analysis:

To provide additional strength to our observation, we did average nucleotide identity (ANI) analysis, which routinely used to find the closeness between the virus/bacterial species including the identification of the species boundary. ANI between all the Sarbecovirus strains was calculated by using OrthoANIu tool [37]. The ANI results show, among all bat and pangolin CoVs, bat RaTG13 share highest ANI (∼96%) with the human SARS-CoV-2s (Figure 4-see PDF). Thus, this observation along with phylogeny based evidences indicates that SARS-CoV-2 is thought to have emerged from Bat coronavirus RaTG13.

## Gene tree analysis:

In addition to the whole genome phylogeny, gene tree analysis was also conducted as it provides a more reliable basis for studying species evolution. Five gene trees namely Orf1ab, Spike, Envelope, Membrane, and Nucleocapsid of the Betacoronaviruses were reconstructed for gene tree analysis (Figure 5 and Figures. S2-S5-see PDF). Except Nucleocapsid genetree (Figure S5-see PDF), other four gene trees have shown that the five subgenera clustered according to their cladistic relations where Embecovirus clade appeared as a basal radiation of the Betacoronavirus gene trees. Further, these gene trees were in concordance with the genome tree. The topological difference of Nucleocapsid gene tree with the Betacoronavirus genome/species tree might be possible as gene tree differs from species tree for various analytical and/or biological reasons [38,39, 40]. Further, analysis on the gene trees found, except Envelope gene tree, other four gene trees exhibited bat-CoV-RaTG13 is the closest relative of SARS-CoV-2 followed by pangolin-CoV as found in the genome tree analysis (Figure 3 and Figure 5-see PDF). Different evolutionary pattern of Envelope gene tree is probably due to stochastic error as its length is very small (average length 75 amino acids) [40]. Further analysis of the gene trees found though subgenera-wise four gene trees are similar, but within subgenera there are widespread phylogenetic incongruence [41]. This result led us to hypothesize that recombination events had occurred among Beta coronaviruses in the past that are caused to evolve new strains including the emergence of pathogenic lineage like SARS-CoV-2.

## Recombination analysis::

 Accordingly, we conducted both genome and gene recombination analysis of the Beta coronaviruses using RDP5 package [17]. The genome recombination analysis detected 21 putative recombination signals (Table 1-see PDF). A recombination event was reported when five out of seven methods detected it. Recombination results show that major recombination events took place between bat coronaviruses belonging to the subgenus Sarbecoviruses. A recent study by Boni et al. (2020) also reported the Serbicoviruses lineage undergoes frequent recombination [42]. For further insights, we compared SARS-CoV-2 Hong Kong (HKU_SZ_005b) genome sequence with four closely related SARS-CoVs namely Bat-CoV-RaGT13, Bat-SL-CoVZC45, Bat-SL-CoVZXC21, and Pangolin-CoV-GX-P5E using simplot analysis (Figure 6-see PDF). Simplot exhibits that bat-CoV-RaTG13 shows the highest similarity with SARS-CoV-2 genome including exchange of genetic materials at the different regions as shown in Figure 6(see PDF). We classified the whole genomes into four regions (Regions1-4). In region 1 (which mostly covers ORF1a gene), we observed highest genetic divergence between pangolin and SARS-CoV-2 strains, and bat to bat recombination events were frequent. In region2 (ORF1b gene), recombination events mostly took place between bat and pangolin strains. In region3 (Spike gene), bat-CoV-RaTG13 genome shows divergence with SARS-CoV-2 genome and there is a good number of genetic recombination among the bat and pangolin strains. In region4 (E, M, N and ORF3/6-8/10 genes), all strains show high similarity and a few number of recombination events with the SARS-CoV-2 strain. Further, gene recombination analysis found that there are highest recombination events in spike protein (spotted nine events) followed by Orf1ab protein (six events). Membrane and Nucleocaspid proteins reported few recombination events and envelope protein did not show any recombination event. Overall, recombination results support our phylogenetic inference and suggest that the origin of SARS-CoV-2 is the results of ancestral intra-species recombination events between bat SARS-CoVs [43,44]. Details of recombination analysis are given in Table 1(see PDF)

Genetic variation analysis Further we measured the genetic variation of bat-CoV-RaTG13 and pangolin-CoV-GX-P5E sequences with respect to SARS-CoV-2 Wuhan-Hu-1 strain, and found that spike protein has highest genetic variation 3% and 7 % respectively (Table 2-see PDF). We further did MSA of the spike protein sequences and observed that the insertion of the novel amino acids "PRRA" in the spike protein of human SARS-CoV-2s (Figure 7-see PDF). The "PRRA" insertion at the S1/S2 junction site which induces a furin cleavage motif needs to be investigated. Therefore, further detailed study on these residues would be required to shed light on molecular mechanism of interaction between SARS-CoV-2 and the host cells.

## Structural analysis of spike protein::

On the basis of MSA result, we compared the structure of spike protein of SARS-CoV-2 (PDB: 6XLU) with bat-CoV-RaTG13 (PDB: 6ZGF) and pangolin-CoV-GX-P5E (modeled protein) (Figure 8-see PDF). The spike protein is a complex trimeric protein and monomer was used for structure comparison. It has two main units S1 and S2. The S1 subunit recognizes and binds to the host receptor enzyme via receptor-binding domains (RBDs) while the S2 subunit helps in fusion of viral cell membrane to host cell [45,46,47]. We found that structurally the spike protein of pangolin-CoV-GX-P5E is more diverse compared to SARS-CoV-2 (RMSD value 2.766 Å) while the bat-CoV-RaTG13 spike protein shows similarity to SARS-CoV-2 with RMSD 2.059 Å (Figure 8-see PDF). It was observed that the bat-CoV-RaTG13 shows high number of mutations at one of the RBD (spotted 27 mutations: shown in green colour in Figure 8-see PDF) while the pangolin-CoV-GX-P5E shows mutations at both the RBDs of S1 subunit (a total of 85 mutations). The changes in spike proteins have impact on the interaction of pathogen and host [48, 49]. Thus these mutations were probably responsible for the adaptation of SARS-CoV-2 into human systems. A number of studies reported that the mutations in spike protein of SARS-CoV-2 facilitate its adaptation into humans [50, 51,52]. The insertion of the four amino acids "PRRA" found in the MSA represents an extended loop between the two parallel β-sheets (S1/S2 cleavage site). This cleavage point between the receptor binding domain (S1) and fusion peptide (S2) mediate cell-cell fusion and entry into human cell [25, 47]. Thus structural analysis supports MSA results and suggests that SARS-Cov-2 is adapted to infect human systems.

## Conclusions:

Outbreak of SARS-CoV-2 is the third documented spillover of an animal coronavirus to humans in only two decades that has resulted in a major pandemic. In quest of the emergence of SARS-CoV-2, this study finds that human SARS-CoV-2 emerged from Bat-CoV-RaTG13 through ancestral intra-species recombination events between bat corona viruses belonging to Sarbecovirus subgenus. Furthermore, acquisition/insertion of a furin cleavage motif("PRRA") to the S1/S2 site in the spike protein of SARS-CoV-2 along with high number of mutations at one of its RBD are probably responsible for the adaptation of SARS-CoV-2 into humans systems. Therefore, further detailed study on these residues would be required to shed light on molecular mechanism of interaction between SARS-CoV-2 and the host cells.
